# Impact of Year-Round Grazing by Horses on Pasture Nutrient Dynamics and the Correlation with Pasture Nutrient Content and Fecal Nutrient Composition

**DOI:** 10.3390/ani9080500

**Published:** 2019-07-29

**Authors:** Sara Ringmark, Anna Skarin, Anna Jansson

**Affiliations:** 1 Department of Anatomy, Physiology and Biochemistry, Swedish University of Agricultural Sciences, SE-75007 Uppsala, Sweden; 2 Department of Animal Nutrition and Management, Swedish University of Agricultural Sciences, SE-75007 Uppsala, Sweden

**Keywords:** pasture, horse nutrition, crude protein, exclosures

## Abstract

**Simple Summary:**

Horse grazing may benefit biodiversity. This study compared the effect of horses grazing year-round to that of mowing on pasture quality in a forest-grassland landscape in Sweden. Twelve Gotlandsruss stallions were kept in three enclosures (~0.35 horse/hectare) without supplementary feeding for 2.5 years. Each enclosure contained three exclosures where pasture was not grazed, but mown monthly. Horse grazing increased the diversity of pasture nutrient content. Moreover, energy and protein concentrations and grass availability increased in areas grazed by horses, but decreased where grass was mown. This indicates that year-round grazing can be used to increase biodiversity, a suggestion supported by botanical observations. Nutrient content in horses’ droppings was found to correlate with nutrient content in pasture, so analysis of droppings may be used to roughly estimate the quality of pasture consumed by horses. Under the conditions studied, pasture protein content was sufficient to meet horse requirements year-round, while energy content and pasture availability may have been limited in winter. Monthly data presented here on the nutritive value of pasture can help guide the management of year-round grazing systems in the Nordic countries.

**Abstract:**

Horse grazing may benefit biodiversity, but the impact of year-round grazing on nutrient dynamics has not been evaluated previously. This study compared pasture quality in a forest-grassland landscape grazed year-round by horses with that in exclosed mown areas. Twelve Gotlandsruss stallions were kept without supplementary feeding in three enclosures (~0.35 horse/ha) outside Uppsala, Sweden, from May 2014 to September 2016. Each enclosure contained three mown exclosures, where grass sward samples were collected monthly and analyzed for chemical composition and vegetation density. Fecal grab samples were collected and analyzed for crude protein (CP) and organic matter (OM) content. There were no differences in exclosure pasture energy or CP content between enclosures (*p* > 0.05). In grazed areas, there were differences in grass energy and CP content (*p* > 0.05) between enclosures. During the three summers studied, energy and CP content increased in the enclosures, but decreased in the exclosures. By the end, biomass content/ha was greater in the enclosures than in the exclosures. Fecal OM and CP content showed moderate to strong correlations with pasture nutrient content (*r* = 0.3–0.8, *p* < 0.05). Thus, in contrast to monthly mowing, horse grazing diversified pasture chemical composition and increased its nutritive value.

## 1. Introduction

Year-round grazing by cattle, sheep, and horses is common in many European countries, but not in Sweden. To our knowledge, the effects on pasture quality and quantity of keeping horses year-round on extensive grazing have not been evaluated previously in the Scandinavian countries. Reasons for this might include the comparatively short growing season, the need for shelter to meet animal welfare legislation, and expected low nutrient content of pasture during winter. Lack of validated methods for monitoring horse nutrient intake on pasture may be another reason. However, studies in Germany have shown that the nutrient content of pastures grazed year-round can meet or exceed the requirements of adult cattle and horses, even in winter [1]. In contrast, a study on year-round grazing horses in France indicated that crude protein intake was very low six months per year, and that adult maintenance requirement was met only in April–September [2]. This estimation was based on fecal analyses of crude protein and an observed positive correlation between dietary and fecal crude content. The use of fecal crude protein analysis to monitor pasture crude protein content and intake needs, however, to be further validated.

Year-round grazing systems may have the potential to reduce feed costs, but may also support horses’ natural behaviors and contribute to increased biological diversity. Abandonment of natural and semi-natural grasslands and forest encroachment, induced by lack of large herbivores in open landscapes, has caused loss of flora and fauna biodiversity in Sweden [3]. Studies in European countries, including Sweden, indicate that grazing horses can be used instead of cattle and sheep to promote biological diversity [4,5,6,7,8,9]. Horses remove more vegetation per unit body mass than cattle [10], create mosaic patches of short and tall grass, and leave more broad-leaved plants than cattle [10]. Horses prefer grasses [2], but their intake of forbs and shrubs may increase during periods of intense grazing in winter and spring [11,12], and they may also perform bark-stripping [13]. Use of horses in a year-round grazing system could therefore have great impacts on the landscape and biological diversity [14].

The overall aim of this study was to describe the seasonal and land-to-land variation in pasture quality in a Swedish forest-grassland landscape grazed year-round by horses, and compare it with that in adjacent exclosure areas mown monthly. A second aim was to investigate fecal sampling as a measure of pasture quality. The results are discussed in relation to whether the fodder quality was acceptable to meet energy and protein requirements in horses, and to the possible impact on pasture diversification. The hypotheses tested were that pasture energy and protein content can meet animal requirements but with differences between land areas; that horse grazing alters pasture energy and nutrient composition compared with mowing; and that fecal crude protein (CP) content is correlated with pasture nutrient concentration.

## 2. Materials and Methods

The study was carried out between May 2014 and September 2016 in Krusenberg, Uppsala, Sweden (59°44′8” N, 17°38′58” E). During the 15 years preceding the study, mean daily temperature April–October was 12.4 ± 5.0 °C (±SD) and mean precipitation was 1.7 ± 4.3 mm/day, while in November–March the values were −0.6 ± 5.4 °C and 1.4 ± 2.7 mm/day, respectively (Swedish Meteorological and Hydrological Institute (SMHI) weather station Uppsala Aut, https://www.smhi.se/klimatdata).

In our study, the summer was defined to start after the first four consecutive days with mean temperature >+5 °C in spring and to end after the first four days with <+5 °C in fall, which defined the start of winter. Based on this definition, the summer season started on 11 April 2015 and on 30 March 2016, while the winter season started on 9 November 2014 and on 10 October 2015. The study was approved by Uppsala animal welfare ethics committee (license number: C28/14). Data on daily temperature and monthly precipitation during the study period were retrieved from the Department of Earth Sciences, Uppsala University, Sweden (www.geo.uu.se).

### 2.1. Horses and Management

**Figure 1:**
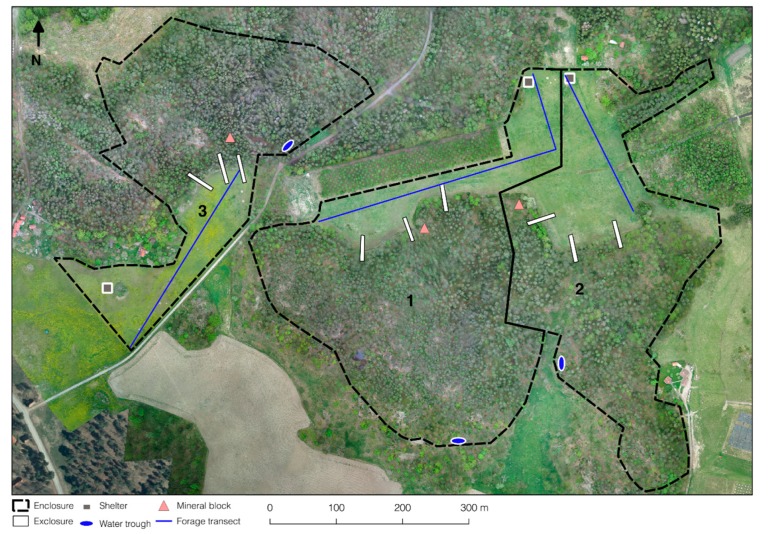
Aerial view of the three enclosures (1, 2, and 3) used in the study, showing position of shelters, water troughs, mineral blocks, exclosures, and pasture transects. Photo taken on 24 May 2016 at 150 m altitude.

Twelve one-year old Gotlandsruss stallions (mean body weight 185 ± 21 kg at the start) from six different breeders were used in the study. Gotlandsruss is a native Swedish horse breed that has been present on the island of Gotland from at least the seventeenth century [15] and probably the thirteenth century. The horses were divided into three groups of four and allocated to three enclosures at the start of the experiment on 21 May 2014. The horses were kept without supplementary feeding throughout the study. To avoid the grazing preferences of an individual horse or group affecting pasture composition, the groups were rotated between the enclosures on 27 May 2015 and 20 May 2016, i.e., each group grazed each enclosure for one growing season. In January 2016, one individual was excluded from the study due to an injury. Each enclosure contained a 16 m^2^ shelter (Figure 1). Water was offered in automatic water troughs, located in the forest, during summer, spring, and fall. During winter, when the temperature was below 0 °C, water was offered once/day in plastic troughs. In all enclosures, water was also available in streams in the forest, even during winter. A salt block with trace minerals (May 2014–August 2014: Ab Hansson & Möhring, Halmstad, Sweden, content (mg/kg): Zinc 300, manganese 200, copper 80, iodine 50, selenium 20, cobalt 12; August 2014–September 2016: Standard, KNC, Netherlands, content (mg/kg): Zinc 810, copper 220, iodine 100, selenium 20) was provided in all enclosures. Horses were dewormed five times during the study period, using Banminth (Pyrantel, Zoetis Finland Oy, Helsinki, Finland), Equimax (Ivermectin and Praziquantel, Sofarimex Indústria Química e Farmacêutica Ltd., Cacém, Portugal), or Cydectin (Moxidectin, Zoetis Manufacturing & Research Spain, Gerona, Spain). Once per month, fresh grab sample (approximately 300 g) of faces were collected immediately after defecation from a minimum of two horses/enclosure (in total a minimum of six samples). These samples were stored at −20 °C until analysis.

### 2.2. Enclosures and Exclosures

The three enclosures (En1–En3) were 13, 11, and 10 ha in size, respectively, and consisted of approximately 1/3 fields and 2/3 forest (Table 1). Each was surrounded by electric fencing. Dominating vegetation types, according to the classification used by the Swedish land survey (lantmateriet.se), were recorded in plots 15 m^2^ placed at 35 m spacing in a grid and located with GPS [16]. The vegetation was then merged into three different vegetation classes: grassland, forest, and semi-forest. Forest was defined as forested areas with >30% crown coverage and semi-forest with 10–30% crown coverage, while grassland had <10% crown coverage. In addition, plant species and species coverage were recorded in a 20 × 20 cm square in the plots (Table 1). The fields had not been grazed by horses for at least 10 years, but En1 and En2 had been grazed by cattle and En3 had been used for production of conserved forage.

In each enclosure, three exclosures each measuring 42.5 m × 5 m were fenced off using electric fencing (Figure 1). All exclosures were placed in the edge zone between forest and open field, with 20 m of the exclosure in the forest and 22.5 m in the open field.

### 2.3. Pasture Sampling

The pasture in the open field areas was sampled in the second week of each month all year round, except when the ground was covered with snow (0–29 cm, December 2014–March 2015, January 2016, and March 2016). Four types of sample were collected: Forage, volume, graze, and exclosure.

Forage samples were collected by clipping a grab sample of vegetation 5 cm from the ground every 10 m along a transect crossing the open fields. In En2 and En3, samples were collected along that one transect, but due to the shape of En1, the sampling line was L-shaped and longer, and samples were collected every 20 m to retrieve the same amount of samples representing the approximate same size of grassland.

Volume samples were taken along the same transects as the forage samples, but every 50 m (100 m in En1), collecting all vegetation 5 cm from the ground within a 30 cm × 30 cm square (0.09 m^2^). The volume samples were placed in plastic bags and weighed later for determination of pasture quantity.

Graze samples were collected in the area where horses were grazing at the time of sampling. Vegetation was cut close to the ground, i.e., the height at which the horses were assumed to graze.

Exclosure samples were taken within a 50 cm × 50 cm quadrat (0.25 m^2^) in the open field part of the exclosures, 2 m from the fence (Figure 1). All vegetation above 5 cm from the ground within the quadrat was collected by mowing with a scissor. Grassland production was assessed by weighing the Exclosure samples, determining the dry matter (DM) content, and calculating the amount of DM per hectare.

In February and April 2016, only graze samples were collected, due to small sample size/no sample for the other three sample types. At all sampling sites except those for the graze samples, grass height was measured using a herbometer (Herbometre, AGRO-Systémes, La membrolle sur Chosille, France), with a 30 cm × 30 cm square plate placed on top of the vegetation. On occasion, horses were observed eating bilberry plants (*Vaccinium myrtillus* L.) and in December 2014, random samples of bilberry, without berries or leaves, were collected at the time of grazing/browsing. All samples were stored at −20 °C until analysis.

### 2.4. Analyses of Chemical Composition

The exclosure samples from the three exclosures within each enclosure were pooled to one sample before analysis of nutrient content. To determine DM content, pasture and feces samples were dried at 60 °C for 24 h and milled in a 1 mm hammer mill (Kamas, Slagy 200 B, Malmö, Sweden). A 2 g subsample was then dried at 103 °C for 16 h. Ash content was determined by incinerating a 2 g sample at 550 °C for 3 h, after which the residue was cooled and weighed. Organic matter (OM) content was calculated by subtracting the content of ash from the DM content. Digestibility coefficient of organic matter (VOS) and metabolizable energy (ME) content were determined in vitro according to Lindgren 163 [17]. The ME content is, however, based on the ME for ruminants, so was adjusted for horses using the following equation derived by Jansson et al. [18]:

ME_horse_ = 1.12(ME_ruminant_) − 1.1

The concentration of neutral detergent fiber (NDF) was determined according to Chai and Uden [19]. Analysis of CP was performed according to Kjeldahl [20], where ammonia nitrogen concentration was determined by direct distillation with a Kjeltec 2460 analyser (Foss, Hilleröd, Denmark) and N content was multiplied by 6.25 to give the CP content. To estimate the amount of digestible CP (dCP) [19], the following equation used was:

dCP = 0.939–31.1/g CP kg/DM

The ratio between digestible CP and ME (RdCPME) was also calculated, since this is an established measure of horse feed quality in Sweden [18]. In samples retrieved in June 2016 (see Table S1), macronutrient concentrations were analyzed by inductively coupled plasma-atomic emission spectroscopy (ICP-AES) using Spectro Flame equipment (SPECTRO Analytical Instruments, Kleve, Germany). Due to small sample size, values for exclosures in En2 and En3 in October 2014 are missing.

### 2.5. Statistical Analyses

All statistical analyses were performed in Statistical Analysis Systems package 9.4 (SAS Institute Inc., Cary, NC, USA). Differences were considered significant at *p* < 0.05. Values presented are least square means (LSmeans) ± standard error (SE).

To study if climate differed significantly between years during the study period, effects of year on air temperature and precipitation were estimated in a mixed model including an interaction between season and year.

Test of differences in pasture nutrient content between samples retrieved in exclosures and the samples retrieved in the enclosures, as well as possible differences between the different enclosure sample types (Forage, Volume, and Graze), were performed using a mixed model, with enclosure as repeated measurement and an effect of interaction between enclosure and time period (year and month). When the *p*-value for the interaction effect between enclosure and time period was <0.1, a separate analysis without the interaction effect was run. If no interaction effect is reported, values refer to the latter analysis. As analysis of differences in pasture nutrient content showed no significant difference between forage and volume samples, these were pooled before further analysis.

To test if different land areas, i.e., enclosures, responded differently in terms of nutrient content, as well as pasture quantity on horse grazing and mowing, a mixed model with enclosure as repeated measurement was used. The same model was also used to test if season (summer/winter) and year affected the nutritional content and pasture quantity. An analysis including the effect of interaction between year and season was also performed.

## 3. Results

Mean air temperature and precipitation did not differ between the three study years (*p* > 0.05). During summer, mean temperature was 13.3 ± 5.8 °C and mean precipitation was 1.8 ± 4.3 mm/day. During winter, the corresponding values were 1.3 ± 5.1 °C and 1.0 ± 2.2 mm/day.

### 3.1. Variation in Pasture Nutrient Content Between Exclosures and Enclosures, and Between Sample Types

The exclosure samples showed lower DM and higher CP and ME contents than the forage and volume samples from the enclosures, but there was no difference in NDF content (Table 2). The graze samples showed higher nutrient contents than the other three sample types (with the exception of energy content in exclosure samples, which was similar) (Table 2). There was a significant interaction between sample type and time period for ME per kg OM and CP as a percentage of OM (*p* < 0.05), where ME and CP remained at a high concentration in the period May–September in the graze samples, while decreasing from August onwards in the other sample types.

The bilberry shrubs sampled in the forest had the following composition: DM 45%, CP 7% of DM, digestible CP 32 g/kg DM, ME 3.2 MJ/kg DM, and OM 97% of DM.

### 3.2. Variation in Pasture Quantity

Mean grass sward height was lower and DM and ME content/ha were higher in enclosures compared with exclosures, but there were also differences between the enclosures (Table 3). During the summer in 2016, grass sward height decreased in both enclosures and exclosures compared with in 2015, but DM and ME content/ha only decreased in exclosures (Table 4).

### 3.3. Variation in Pasture Nutrient Content between Enclosures

In exclosure samples, there was no general effect of the different enclosures on any of the nutritional parameters analyzed (Table 5). In the pooled forage + volume samples and in graze samples, the content of ME, CP, and NDF/kg OM showed differences (*p* < 0.05) between enclosures (Table 5).

### 3.4. Variation in Pasture Quality Between Years, Seasons, and Months

**Figure 2:**
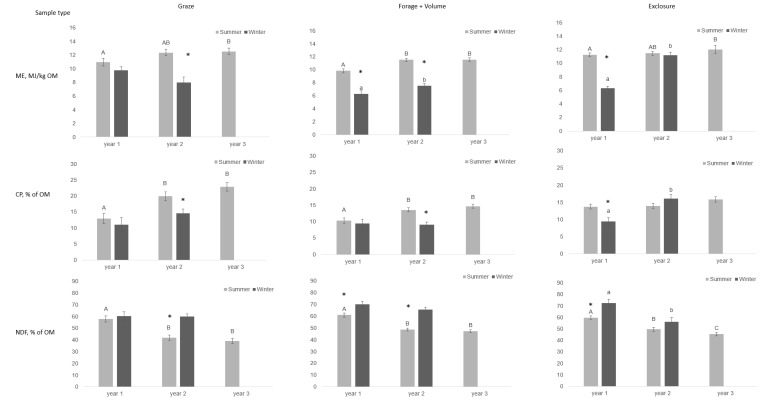
Content of metabolizable energy (ME) per kg organic matter (OM), crude protein (CP) as % of OM, and neutral detergent fiber (NDF) as % of OM in three types of pasture samples (graze, forage + volume, exclosure) collected monthly in three enclosures grazed by 12 Gotlandsruss between May 2014 and September 2016. One year is defined as start of summer season to end of winter season. Each enclosure contained three exclosures. An asterisk indicates significant difference between seasons within year (*p* < 0.05). Different lowercase letters (a, b) indicate differences between years within winter season, while different uppercase letters (A, B) indicate differences between years within summer season (*p* < 0.05).

As pasture was not sampled in all months throughout the year, only differences within seasons between years are presented (Figure 2). During the study period, summer pasture NDF concentration decreased in the enclosures, while ME and CP concentrations increased (Figure 2). In the exclosures, summer pasture CP remained unchanged between years, while ME concentration increased and NDF concentration showed a decrease.

Mean monthly nutrient composition and RdCPME in forage + volume and graze samples in all three enclosures during the whole study period are shown in Tables S2 and S3. Mean ME content ranged from 4.9 ± 0.5 to 12.0 ± 0.6 MJ per kg DM, mean CP content ranged from 7 ± 1 % to 24 ± 3 %, mean digestible CP per kg DM ranged from 37 ± 12 g to 190 ± 26 g, and mean RdCPME ranged from 6.4 ± 1.1 to 15.9 ± 2.6 (Table S2).

### 3.5. Fecal Composition and Correlation with Pasture Nutrient Content

**Figure 3:**
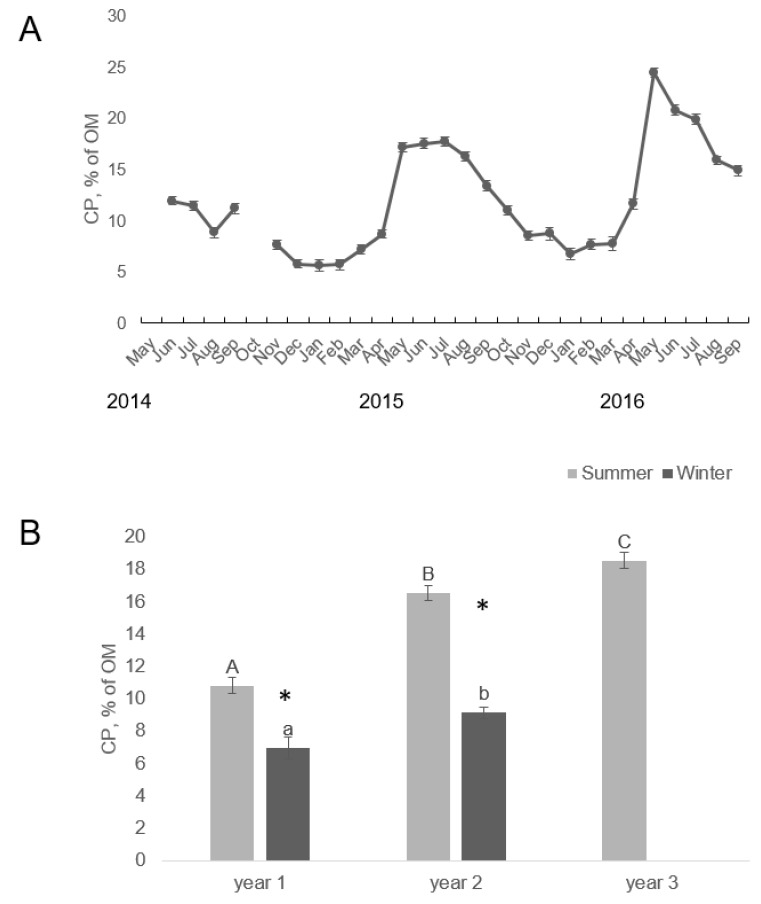
(**A**) Monthly variation and (**B**) seasonal and between-year variation in crude protein (CP) content (LSmeans ± SE) as % of organic matter (OM) in feces from Gotlandsruss grazing without supplementary feeding from May 2014 to September 2016. One year is defined as start of summer season to end of winter season.

The OM and CP content in feces, but not the DM content, were dependent on the individual horse, but overall the OM content in feces was lower in En3 than in En1 and En2 (Table 6). Content of CP, both as % of OM and as % of DM, was lowest in En1 and highest in En3 (Table 6). Concentrations of CP in feces were lower in winter than in summer (Figure 3). Within season, fecal CP concentration increased with year.

The content of DM, OM, and CP in feces showed moderate to strong correlations with the DM, NDF, OM, and CP concentrations in graze samples and in forage + volume samples (Table 7 and Table 8).

## 4. Discussion

The results obtained in this three-year study showed that horse grazing altered pasture nutrient composition and diversified pasture chemical composition (between enclosures) to a greater extent than mowing. This indicates that horses can manage pasture and are therefore suitable for year-round grazing in Sweden, as a means to increase pasture diversity. To our knowledge, this is the first study at Nordic latitudes to evaluate the effect on pasture chemical composition of year-round grazing by horses without supplementary feeding. Year-round grazing is currently not practiced in the region because of the lack of vegetation growth in winter (i.e., temperatures below 5 °C for more than four days). However, the study area is within a zone suggested to be suitable with respect to climate conditions for rewilding of horses, although local biotic factors, land cover, and soil type may influence the degree of suitability [21]. The study was conducted between 2014 and 2016, under temperature and precipitation conditions typical for the region, and the results are therefore of general relevance for this form of horse management.

### 4.1. Pasture Quality and Effects of Sample Type

One likely explanation for the diversified nutrient content of grazed pasture is the grazing behavior of horses. Horses perform selective grazing [22,23], and also create mosaic landscape patterns [24], as some areas are frequently grazed close to the ground while others are avoided. In the present study, this was reflected in the higher content of ME and CP and lower content of NDF in the graze samples, compared with the forage and volume samples, indicating an earlier botanical stage. An additional explanation for the altered nutrient content may be the change in botanical composition reported previously for the study area [4]. For example, grazing favored prostrate plant species (low plant height at maturity).

The three enclosures all had different qualities. En1 and En2 showed a different response to En3 with respect to sward height and nutrient content, for example. The reason is unclear, but En3 had previously been cultivated (forage production) and En1 and En2 had been grazed by cattle. En2 was also the enclosure with the lowest grazing pressure, as it had a larger grass area (3.3 ha, compared with 2.7 ha in En1 and En3), and the plant composition differed (Table 1). Mowing of the exclosures was performed without selection, creating similar plant stress between enclosures, which might have evened out initial differences in species and chemical composition. Horses, on the other hand, graze selectively [22,23] and might have favored different species and areas in the three enclosures, as well as putting more stress on the plants by grazing them shorter than mowing (i.e., <5 cm). In addition, the trampling effect of horses might have affected the botanical composition and, therefore, also the nutrient content.

Compared with the exclosure areas, from which grazing horses were excluded, grazed pasture showed both lower (volume and forage samples) and higher (graze samples) concentrations of CP, depending on sample type. The difference in composition between sample types may be due to the forage + volume samples and the exclosure samples including only plant parts >5 cm. These plant parts may be of a later botanical stage [25], as reflected by the higher content of NDF compared with graze samples collected close to the ground. Both the graze and forage + volume samples showed relatively high correlations with fecal NDF and CP content, indicating that they may both be valid sampling options when measuring the nutrient content of pasture consumed by horses.

Observations from Sweden indicate that heavily grazed grasses may have higher CP and energy content in October than grasses subjected to lower grazing pressure [26]. This is likely due to old biomass being replaced by new, nitrogen-rich leaves as grazing increases defoliation [27], and is presumably the reason for the higher nitrogen content in the graze samples in the present study. During summer seasons, ME and CP increased in graze and forage + volume samples, while they were unchanged in exclosure samples and remained high in the graze samples for longer than in the other sample types. These results are in accordance with observations in sheep pastures that frequent grazing during the vegetation period improves nutrient quality compared with mowing [28]. The results confirm that grazing managed at the right intensity can enhance the quality of pasture [29].

### 4.2. Pasture Quantity

Pasture dry matter production in the study area was within the range reported for other horse and ruminant year-round grazed pastures in Germany [1], and also similar to that reported for natural pastures in Sweden grazed by cattle and sheep [30]. In addition, based on the 10-year mean for forage harvest from cultivated grassland, the years included in the study seem to be representative for the region [31].

The pasture quantity values determined in the exclosures could be regarded as a measure of overall pasture production, while the volume samples collected in the enclosures could be considered a measure of the amount of pasture available to the horses. The exclosures were mown at the same sites as the exclosure samples were taken. In contrast, the volume samples were collected by walking along a straight line, and with this method, sampling sites may have varied slightly between sampling occasions. Comparisons of pasture and energy quantity between enclosures and exclosures should therefore be made with caution. However, the lack of differences in grass sward height between enclosures and exclosures implies that mowing to 5 cm every month resulted in a similar rate of vegetation removal as horse grazing within the enclosures.

Pasture quantity decreased over the study years in both enclosures and exclosures (2015 and 2016). This may therefore be an effect of annual variation, rather than an effect of horse grazing, on pasture production. To evaluate the long-term impact of horse grazing on pasture production, much longer studies are required.

### 4.3. Ability of Pastures to Meet the Nutritional Requirements of Horses

Despite the cold winters in the region, CP content during winter (7–11% of DM) was within the range reported for other European winter pastures [1]. Assuming a maximum DM intake capacity of 3% of body weight [32,33,34], a 250 kg adult stallion with a CP requirement of 432 g/day [35] would, in theory, manage with a CP concentration in pasture of 6% of DM, which is just below the lowest value recorded in the present study. However, as found for other European pastures [1], the amount of pasture available and the ME content during winter were insufficient to maintain body condition in horses. In addition, a snow layer of >10 cm covered the ground for 14–31 days/winter in our study, making the pasture more difficult to access. At the beginning of winter, when snow was still absent, the mean energy content of the pasture was estimated to be 6200 MJ ME/ha in the grasslands. Assuming a winter season lasting five months, and that all vegetation sampled here could be consumed by the horses, this would supply each horse with 10 MJ ME/day, which represents 30% of the estimated daily requirements [35]. However, in reality the samples would probably contain plants generally not consumed by the horses and the energy requirement of the horses may have been higher during cold spells.

The insufficient levels of energy and CP in the pasture were reflected in loss of body weight and body condition in the horses during the winter months (unpublished data). On the other hand, during the growing season, pasture provided a surplus of energy great enough for the horses to store body fat, compensating for energy deficiency in winter. En2 was the only enclosure where no horse at any time would have required supplementary feeding to maintain a functional body condition. An energy content in pasture of at least 12,000 MJ ME/ha in November therefore seems sufficient to avoid horses becoming underweight at the given animal density (approximately 1000 kg horse on 3 ha of grassland and 7 ha forest) and summer conditions. However, the lack of need for supplementary feeding in En2 could also be due to this enclosure containing a rather large area (1.3 ha) of semi-forest with some grass in the understory.

Interestingly, during wintertime, horses spent more time in the forest (unpublished data). Therefore, tree materials, some grass, and bilberry plants probably comprised a greater proportion of the horses’ diet during winter. The chemical analysis of bilberry plants showed rather low contents of ME and CP. However, it should be noted that the analytical methods used are designed for grasses and legumes, and may be less relevant for shrubs.

### 4.4. Correlation of Pasture Quality and Fecal Composition

The correlations between fecal and pasture concentrations of CP (as % of DM and OM) were moderate to strong (*r* = 0.57–0.67). These were similar to correlations reported for stabled horses fed a forage-only diet [36]. The fairly high correlation implies that a fecal grab samples could be used to give a rough estimate of CP intake in grazing horses. However, the method may not apply if the horses are growing or mares are lactating, as their nutrient requirements are higher than those of adult horses [35].

The trend for fecal CP concentration to be correlated with season is similar to that reported for bachelor horses in Camargue [2] and feral horses in Canada [37]. However, the seasonal variation in the present study was greater, ranging from 5.6% of DM in January to 17.0% of DM in May. The increment in fecal CP content with year reflected the pasture composition, as CP content in pasture vegetation was also higher in the second and third summer than at the start of the study.

Surprisingly, the correlation with fecal CP concentration was slightly stronger with forage + volume samples than with graze samples. The highest CP values were observed in Graze samples and the CP content was periodically much higher than the requirement. However, this was not reflected in higher CP content in feces compared with the forage + volume samples, probably because more of the easily digestible nitrogen was excreted with urine and not with feces [38]. The intention with collecting the graze samples was to get a more accurate estimate of the nutritional composition of the pasture actually consumed by the horses. However, both the graze samples and the grab sample of the feces were spot-samples taken at random times and the graze samples represented conditions at those times, while fecal samples would consist of digesta ingested hours to days before sampling [38]. This may be another reason why the CP content of forage + volume samples correlated better with CP concentration in feces.

### 4.5. Practical Implications

Our study provides practical data on the quality and quantity of Swedish pasture grazed year-round by horses. The area of semi-natural pastures in Sweden is decreasing [39]. At the same time, the number of horses in Sweden is increasing, from 85,000 in 1970 [40] to now approaching 355,000, which makes horses more common than dairy cows [31]. There is, thus, great potential for using horses in landscape conservation in Sweden. However, most horses are stabled for most of the year and the main roughage fed is hay or haylage harvested from cultivated leys. The results in the present study indicate that pasture grazed year-round south of latitude 60° N in Sweden can have a sufficient energy and nutrient content to meet the nutritional recommendations of adult horses for at least 10 months per year (Table S3, no data for January and March) and that the amount of pasture may be a limitation. The results presented here could be used as the basis for recommendations on utilization of semi-natural pastures by horses even outside the growing season, as an alternative to feeding hay or haylage, for example. Compared with feeding conserved forage, pasture provides increased opportunities for horses to express their natural behavior and requires less resources than harvesting, conservation, and transportation of hay/haylage. The low content of energy in shrubs such as bilberry means that forest pastures could be suitable to meet the feeding behavior requirement of obese horses, although this would require further evaluation. Increased grazing of semi-natural pastures would also increase biological diversity [4] and help preserve agricultural landscapes. Moreover, our data on the nutrient composition of Swedish semi-natural pastures support the suggestion [21] that they could be suitable for future rewilding of horses at this latitude.

## 5. Conclusions

Compared with mowing, year-round grazing by horses in Sweden increased pasture nutrient quality and diversity. This indicates that year-round grazing by horses in Sweden could be used as a general tool to increase biodiversity. Pasture sampling method affected the pasture quality results but, overall, CP content was sufficient to meet the horses’ requirements year-round, while the energy content and pasture availability may be a limitation during winter. Fecal grab samples proved to give a fairly good estimate of CP intake in grazing horses, but should be complemented with analysis of pasture quality for pregnant, lactating, and young horses with high CP requirements.

## Figures and Tables

**Table 1:** Area (ha) of vegetation types within each enclosure and dominant plant species identified in a vegetation inventory performed in all three enclosures in May 2014 (study start).

Vegetation Type, ha	Enclosure 1	Enclosure 2	Enclosure 3
Grassland	2.7	3.3	2.7
Forest	10.7	5.8	6.8
Semi-forest ^a^	0	1.3	0.2
Total area	13.4	10.3	9.7
**Dominant Plant Species in Grassland, %**			
Grasses	60	57	26
Yarrow (*Achillea millefolium*)	10	14	
Dandelion (*Taraxacum* spp.)	7	10	47
Hempnettle (*Galeopsis tetrahit*)		6	
White clover (*Trifolium repens*)			5
**Dominant Ground Cover in Forest, %**			
Mosses	46	29	55
Grasses	11	26	12
Bilberry (*Vaccinium myrtillus*)	12	7	10
Lingonberry (*Vaccinium vitis-idaea*)	5	4	3

^a^ Semi-forest includes forest areas dominated by deciduous forest with a large proportion of grass in the ground cover.

**Table 2:** Content of dry matter (DM), metabolizable energy (ME) per kg organic matter (OM), crude protein (CP) as % of OM, and neutral detergent fiber (NDF) as % of OM in three different types of pasture samples collected in three enclosures grazed by horses, and in three exclosures per enclosure, monthly between May 2014 and September 2016, except for December 2014–March 2015 and January-April 2016.

Sample	Enclosures			Exclosures	*p*
	Forage	Volume	Graze		
ME, MJ/kg OM	10.0 ± 0.2 ^a^	10.0 ± 0.2 ^a^	10.9 ± 0.2 ^b^	10.5 ± 0.2 ^b^	0.0002
CP, % of OM	11.9 ± 0.4 ^a^	11.6 ± 0.5 ^a^	17.0 ± 0.4 ^b^	13.7 ± 0.4 ^c^	<0.0001
NDF, % of OM	57.2 ± 0.7 ^a^	56.7 ± 0.9 ^a^	51.0 ± 0.7 ^b^	56.8 ± 0.8 ^a^	<0.0001
DM, %	34 ± 1 ^a^	34 ± 1 ^a^	29 ± 1 ^b^	27 ± 1 ^b^	<0.0001

^a,b,c^ Different superscript letters indicate significant differences within rows (*p* < 0.05).

**Table 3:** Mean grass sward height and content of dry matter (DM) and metabolizable energy (ME) in the enclosures grazed by horses and in the exclosures within each enclosure mown monthly from May 2014 to September 2016. Exclosures were mown at the same spots as sward height measurements were made.

	Enclosures				Exclosures				*p* Means
	En1	En2	En3	Mean	En1	En2	En3	Mean	
**Grass sward height, cm**	5.5 ± 0.1 ^a^	7.7 ± 0.2 ^b^	5.5 ± 0.2 ^a^	5.3 ± 0.1	4.3 ± 0.4 ^a^	4.9 ± 0.4 ^a,b^	6.0 ± 0.4 ^b^	5.6 ± 0.4	<0.05
**DM, kg/ha**	957 ± 114 ^a^	1393 ± 114 ^b^	741 ± 114 ^a^	886 ± 73	525 ± 111	487 ± 111	721 ± 111	326 ± 76	<0.0001
**ME, MJ/ha**	8494 ± 895 ^a^	11,204 ± 928 ^b^	7351 ± 895 ^a^	7470 ± 636	5208 ± 1171	4912 ± 1171	7760 ± 1171	3048 ± 701	<0.0001

^a,b,c^ Different superscript letters indicate significant differences (*p* < 0.05) between enclosures (En1, En2, En3).

**Table 4:** Mean summer season grass sward height and content of dry matter (DM) and metabolizable energy (ME) per year in the enclosures grazed by horses and in the exclosures within each enclosure mown monthly from May 2014 to September 2016. Exclosures were mown at the same spots as sward height measurements were made.

	Enclosures			Exclosures		
	2014	2015	2016	2014	2015	2016
**Grass sward height, cm**	8.9 ± 0.21 ^a^	5.9 ± 0.2 ^b^	5.2 ± 0.2 ^c^	-	7.1 ± 0.4 ^a^	5.4 ± 0.4 ^b^
**DM, kg/ha**	1560 ± 160 ^a^	863 ± 176 ^b^	770 ± 176 ^b^	714 ± 96 ^a^	760 ± 105 ^a^	260 ± 105 ^b^
**ME, MJ/ha**	13,858 ± 1293 ^a^	8133 ± 1416 ^b^	8176 ± 1466 ^b^	7201 ± 1044 ^a^	7869 ± 1144 ^a^	2810 ± 1144 ^b^

^a,b,c^ Different superscript letters indicate significant differences between years (*p* < 0.05).

**Table 5:** Content of dry matter (DM), metabolizable energy (ME) per kg organic matter (OM), crude protein (CP) as % of OM, and neutral detergent fiber (NDF) content as % of OM in four types of pasture samples collected in three enclosures (En1–En3) grazed all year round by Gotlandsruss and in three exclosures per enclosure. Samples were collected monthly between May 2014 and September 2016, except for December 2014–March 2015 and January–April 2016. LSmeans ± SE, *p*-values indicate the general effect of enclosure.

Sample Type	En1	En2	En3	*p*
Exclosure				
ME, MJ/kg OM	10.8 ± 0.2	10.9 ± 0.2	11.2 ± 0.2	0.2925
CP, % of OM	14.9 ± 0.4	14.3 ± 0.4	13.9 ± 0.4	0.1955
NFD, % of OM	55.0 ± 1.1 ^a^	54.7 ± 1.1 ^a,b^	51.8 ± 1.1 ^b^	0.0924
DM, %	27 ± 1	29 ± 1	26 ± 1	0.2722
Enclosure Graze				
ME, MJ/kg OM	10.4 ± 0.2 ^a^	10.7 ± 0.2 ^a^	11.5 ± 0.2 ^b^	0.0029
CP, % of OM	16.9 ± 0.8 ^a,b^	16.0 ± 0.8 ^a^	18.8 ± 0.8 ^b^	0.0427
NFD, % of OM	52.7 ± 1.4 ^a^	53.9 ± 1.3 ^a^	45.6 ± 1.3 ^b^	0.0001
DM, %	30 ± 1 ^a,b^	31 ± 1 ^a^	27 ± 1 ^b^	0.0159
Enclosure Forage + Volume				
ME, MJ/kg OM	10.1 ± 0.1 ^a^	9.3 ± 0.1 ^b^	10.6 ± 0.1 ^c^	<0.0001
CP, % of OM	11.5 ± 0.4 ^a^	11.9 ± 0.4 ^a,b^	12.5 ± 0.4 ^b^	0.1405
NFD, % of OM	54.7 ± 1.1 ^a^	59.9 ± 1.1 ^b^	54.2 ± 1.1 ^a^	0.0007
DM, %	34 ± 1 ^a,b^	37 ± 1 ^a^	31 ± 1 ^b^	0.0083

^a,b,c^ Different superscript letters within rows indicate significant differences (*p* < 0.05) between enclosures (En1, En2, En3).

**Table 6:** Content of dry matter (DM), organic matter (OM), and crude protein (CP) as % of DM and as % of OM in feces from Gotlandsruss, divided equally between three enclosures (En1–En3) and grazing all year round.

	En1	En2	En3	*p*
**DM, %**	20 ± 0.3 ^a^	19 ± 0.3 ^b^	21 ± 0.3 ^a^	<0.0001
**OM, %**	79 ± 0.5 ^a^	80 ± 0.5 ^a^	75 ± 0.5 ^b^	<0.0001
**CP, % of DM**	8.4 ± 0.2 ^a^	9.1 ± 0.2 ^b^	9.7 ± 0.2 ^c^	<0.0001
**CP, % of OM**	10.8 ± 0.3 ^a^	11.5 ± 0.3 ^b^	13.1 ± 0.3 ^c^	<0.0001

**Table 7:** Correlation coefficient (*r*, *p*-value) between nutrient concentrations in graze samples and in fecal samples from 12 Gotlandsruss kept in three enclosures between May 2014 and September 2016 without supplementary feeding. DM = dry matter, OM = organic matter, CP = crude protein, VOS = digestibility coefficient of organic matter, NDF = neutral detergent fiber.

Graze Samples		Fecal Samples			
		DM	OM	CP, % of DM	CP, % of OM
DM	*r*	0.37703	ns	−0.30271	−0.28796
	*p*	0.0032		0.0198	0.0270
VOS	*r*	−0.58502	−0.32107	0.62955	0.64978
	*p*	<0.0001	0.0132	<0.0001	<0.0001
NDF	*r*	0.36083	0.53560	−0.69221	−0.75195
	*p*	0.0050	<0.0001	<0.0001	<0.0001
OM	*r*	−0.41346	0.36627	ns	ns
	*p*	0.0011	0.0043		
CP, % of DM	*r*	−0.35722	−0.48196	0.60511	0.66090
	*p*	0.0055	0.0001	<0.0001	<0.0001
CP, % of OM	*r*	ns	−0.55235	0.49911	0.57349
	*p*		<0.0001	<0.0001	<0.0001
NDF, % of OM	*r*	0.48350	0.46697	−0.75952	−0.80312
	*p*	0.0001	0.0002	<0.0001	<0.0001

**Table 8:** Correlation coefficient (*r*, *p*-value) between nutrient concentrations in forage + volume samples and in fecal samples from 12 Gotlandsruss kept in three enclosures between May 2014 and September 2016 without supplementary feeding. DM = dry matter, OM = organic matter, CP = crude protein, VOS = digestibility coefficient of organic matter, NDF = neutral detergent fiber.

Volume + Forage Samples	Fecal Samples				
		DM	OM	CP, % of DM	CP, % of OM
DM, %	*r*	0.58646	ns	−0.45184	−0.44157
	*p*	<0.0001		0.0003	0.0005
VOS	*r*	−0.62653	−0.44629	0.75445	0.76465
	*p*	<0.0001	0.0004	<0.0001	<0.0001
NDF, %	*r*	0.57023	0.48945	−0.84249	−0.86310
	*p*	<0.0001	<0.0001	<0.0001	<0.0001
OM, %	*r*	ns	ns	ns	ns
	*p*				
CP, % of DM	*r*	−0.38627	−0.43487	0.64359	0.67240
	*p*	0.0025	0.0006	<0.0001	<0.0001
CP, % of OM	*r*	−0.38872	−0.42891	0.64552	0.67353
	*p*	0.0023	0.0007	<0.0001	<0.0001
NDF, % of OM	*r*	0.56563	0.50105	−0.84228	−0.86426
	*p*	<0.0001	<0.0001	<0.0001	<0.0001

## Supplementary Materials

The following are available online at https://www.mdpi.com/2076-2615/9/8/500/s1: Table S1. Content of macronutrients in pasture; Table S2. Yearly variation in pasture nutrient content in forage + volume samples; Table S3. Yearly variation in pasture nutrient content in graze samples.
