# Supplementary material for: Impact of Year-Round Grazing by Horses on Pasture Nutrient Dynamics and the Correlation with Pasture Nutrient Content and Fecal Nutrient Composition
Source: Animals (Basel). 2019 Jul 29;9(8):500. doi: 10.3390/ani9080500 (PMC6720502; doi:10.3390/ani9080500)
Supplement: Supplementary file 1 [file animals-09-00500-s001.zip › Table S2 VolForNutr.docx]

**Table S2.** Dry matter (DM) content and content per kg DM of organic matter (OM), digestibility coefficient of organic matter (VOS), neutral detergent fiber (NDF), metabolizable energy (ME), crude protein (CP), digestible crude protein (dCP), and dCP:ME ratio in Forage+Volume pasture samples collected monthly in three enclosures grazed year-round by Gotlandsruss. Means ± SE for three enclosures and 2.5 years (May 2014-September 2016). Plants were sampled 5 cm from the ground every 10-20 m. In January-March, there was no available vegetation or there was snow cover on the ground, so samples from this period are missing

|  |  | **DM, %** | |  | **OM, %** | |  | **VOS, %** | |  | **NDF, %** | |  | **ME, MJ** | |  | **CP, %** | |  | **dCP, g** | |  | **Quote dCP/ME** | |
| --- | --- | --- | --- | --- | --- | --- | --- | --- | --- | --- | --- | --- | --- | --- | --- | --- | --- | --- | --- | --- | --- | --- | --- | --- |
| **Month** | **Year^1^** | **mean** | **range** |  | **mean** | **range** |  | **mean** | **range** |  | **mean** | **range** |  | **mean** | **range** |  | **mean** | **range** |  | **mean** | **range** |  | **mean** | **range** |
| **January** |  | - | - |  | - | - |  | - | - |  | - | - |  | - | - |  | - | - |  | - | - |  | - | - |
| **February** |  | - | - |  | - | - |  | - | - |  | - | - |  | - | - |  | - | - |  | - | - |  | - | - |
| **March** |  | - | - |  | - | - |  | - | - |  | - | - |  | - | - |  | - | - |  | - | - |  | - | - |
| **April** | 2015 | 76 ± 2 | 74-78 |  | 92 ± 1 | 91-92 |  | 53 ± 4 | 47-55 |  | 65 ± 4 | 62-69 |  | 5.0 ± 0.6 | 4.3-5.4 |  | 7 ± 1 | 6-9 |  | 37 ± 12 | 26-50 |  | 7.3 ± 1.7 | 6.1-9.2 |
|  | 2016 |  |  |  |  |  |  |  |  |  |  |  |  |  |  |  |  |  |  |  |  |  |  |  |
| **May** | 2015 | 29 ± 7 | 19-37 |  | 91 ± 1 | 90-92 |  | 91 ± 10 | 75-100 |  | 41 ± 13 | 22-59 |  | 10.9 ± 1.4 | 8.7-12.2 |  | 17 ± 4 | 12-21 |  | 129 ± 33 | 78-165 |  | 11.7 ± 2.2 | 8.2-14.1 |
|  | 2016 |  |  |  |  |  |  |  |  |  |  |  |  |  |  |  |  |  |  |  |  |  |  |  |
| **June** | 2014 | 26 ± 4 | 18-32 |  | 91 ± 1 | 90-93 |  | 92 ± 5 | 85-98 |  | 42 ± 7 | 34-54 |  | 11.3 ± 0.7 | 9.8-11.9 |  | 12 ± 2 | 9-17 |  | 84 ± 22 | 51-125 |  | 7.5 ± 1.9 | 4.7-11.2 |
|  | 2015 |  |  |  |  |  |  |  |  |  |  |  |  |  |  |  |  |  |  |  |  |  |  |  |
|  | 2016 |  |  |  |  |  |  |  |  |  |  |  |  |  |  |  |  |  |  |  |  |  |  |  |
| **July** | 2014 | 31 ± 6 | 25-39 |  | 90 ± 1 | 90-92 |  | 84 ± 6 | 76-91 |  | 48 ± 7 | 39-59 |  | 10.0 ± 0.8 | 8.8-11.2 |  | 11 ± 3 | 7-15 |  | 71 ± 24 | 36-106 |  | 7.1 ± 2.0 | 3.9-9.5 |
|  | 2015 |  |  |  |  |  |  |  |  |  |  |  |  |  |  |  |  |  |  |  |  |  |  |  |
|  | 2016 |  |  |  |  |  |  |  |  |  |  |  |  |  |  |  |  |  |  |  |  |  |  |  |
| **August** | 2015 | 35 ± 4 | 29-38 |  | 90 ± 1 | 89-93 |  | 79 ± 6 | 71-87 |  | 51 ± 7 | 44-63 |  | 9.1 ± 0.9 | 7.8-10.3 |  | 10 ± 1 | 8-11 |  | 59 ± 10 | 43-70 |  | 6.4 ± 0.7 | 5.0-7.3 |
|  | 2016 |  |  |  |  |  |  |  |  |  |  |  |  |  |  |  |  |  |  |  |  |  |  |  |
| **September** | 2014 | 35 ± 6 | 25-41 |  | 90 ± 1 | 89-91 |  | 76 ± 6 | 65-82 |  | 53 ± 3 | 49-58 |  | 8.5 ± 0.9 | 6.6-9.3 |  | 9 ± 1 | 8-11 |  | 56 ± 6 | 48-71 |  | 6.7 ± 1.0 | 5.2-8.3 |
|  | 2015 |  |  |  |  |  |  |  |  |  |  |  |  |  |  |  |  |  |  |  |  |  |  |  |
|  | 2016 |  |  |  |  |  |  |  |  |  |  |  |  |  |  |  |  |  |  |  |  |  |  |  |
| **October** | 2014 | 34 ± 12 | 18-53 |  | 90 ± 1 | 89-91 |  | 69 ± 4 | 63-74 |  | 58 ± 4 | 51-62 |  | 7.5 ± 0.7 | 6.5-8.3 |  | 8 ± 1 | 7-10 |  | 48 ± 9 | 36-58 |  | 6.4 ± 1.1 | 4.5-7.3 |
|  | 2015 |  |  |  |  |  |  |  |  |  |  |  |  |  |  |  |  |  |  |  |  |  |  |  |
| **November** | 2014 | 32 ± 13 | 22-52 |  | 90 ± 1 | 89-92 |  | 62 ± 9 | 53-77 |  | 62 ± 6 | 54-68 |  | 6.5 ± 1.3 | 5.1-8.8 |  | 9 ± 3 | 6-14 |  | 55 ± 28 | 27-102 |  | 8.5 ± 3.6 | 4.0-14.0 |
|  | 2015 |  |  |  |  |  |  |  |  |  |  |  |  |  |  |  |  |  |  |  |  |  |  |  |
| **December** | 2015 | 41 ± 4 | 37-44 |  | 90 ± 0 | 89-90 |  | 58 ± 6 | 51-64 |  | 59 ± 2 | 58-61 |  | 5.9 ± 1.0 | 4.8-6.8 |  | 8 ± 1 | 7-9 |  | 46 ± 10 | 36-55 |  | 7.8 ± 1.2 | 6.8-9.1 |

**^1^**Year(s) in which samples were collected.
