# Supplementary material for: Impact of Year-Round Grazing by Horses on Pasture Nutrient Dynamics and the Correlation with Pasture Nutrient Content and Fecal Nutrient Composition
Source: Animals (Basel). 2019 Jul 29;9(8):500. doi: 10.3390/ani9080500 (PMC6720502; doi:10.3390/ani9080500)
Supplement: Supplementary file 1 [file animals-09-00500-s001.zip › Table S1 Pasture contents of macro minerals.docx]

**Table S1.** Content of macronutrients (g/kg dry matter) in pasture samples taken in June 2016

| Enclosure, sample | Ca | K | Mg | Na | P | S |
| --- | --- | --- | --- | --- | --- | --- |
| En1 Graze | 9.4 | 24.2 | 2.0 | < 0.1 | 2.8 | 2.0 |
| En1 Forage+Volume | 7.5 | 25.9 | 1.8 | < 0.1 | 2.3 | 1.5 |
| En1 Exclosures | 8.5 | 25.4 | 2.0 | < 0.1 | 2.4 | 2.1 |
| En2 Graze | 5.4 | 27.4 | 1.6 | < 0.1 | 1.8 | 3.6 |
| En2 Forage+Volume | 5.8 | 26.2 | 1.6 | < 0.1 | 2.0 | 2.2 |
| En2 Exclosures | 6.7 | 24.8 | 1.8 | < 0.1 | 2.0 | 1.6 |
| En3 Graze | 12.8 | 20.4 | 2.8 | 0.8 | 2.3 | 1.7 |
| En3 Forage+Volume | 8.4 | 34.1 | 1.9 | < 0.1 | 2.9 | 1.4 |
| En3 Exclosures | 7.1 | 23.5 | 2.2 | < 0.1 | 2.4 | 1.4 |
